# A comprehensive bibliometric analysis of research on health and environmental impacts of particle emissions from wood combustion in residential heating

**DOI:** 10.1007/s11356-025-36736-2

**Published:** 2025-07-17

**Authors:** Marie Khedari, Audrey Villot, Olli Sippula, Pasi Jalava, Yves Andres

**Affiliations:** 1 Department of Energy Systems and Environment, GEPEA, UMR CNRS 6144, IMT Atlantique, Nantes, 44307 France; 2 Department of Environmental and Biological Sciences, University of Eastern Finland, Yliopistonranta 1, P.O. Box 1627, 70210 Kuopio, Finland

**Keywords:** Bibliometric review, Environmental impact, Fine particles, Health impact, Residential heating, Wood combustion

## Abstract

Wood is the first and continues to be an important renewable energy source in residential heating. Transition to wood-based energy production has been supported by countries due to climate policies and rising costs of other energy sources. However, small-scale wood combustion releases harmful pollutants, including fine particles, some of which have undefined adverse health and environmental impacts. Researchers used different methods to assess the harmful effects of wood combustion. Since this is a multidisciplinary topic with various interconnected areas, our objective is to conduct a bibliometric analysis to highlight the main research axis and its significance. It is expected that it could prominently contribute to the comprehensive understanding of knowledge in this field and pave the way for future research directions. To this end, a visual-quantitative analysis approach with the identification of publication directions over the last two decades is adopted. The related scientific literature was drawn from Scopus and an in-depth scientific mapping analysis was performed. Our bibliometric results highlight that residential wood combustion (RWC) poses serious risks to human health and contributes to climate change, including decreased life expectancy, elevated cancer risk, and escalated global temperatures. Emerging research includes human health-related risk assessment, brown carbon light absorption, and chemical composition. The lack of a link between health effects and chemical composition of particle emissions was observed. Therefore, future research should aim to implement an integrated assessment of RWC particles’ properties, alongside the parallel development of emission-health risk factors, to link emissions with impacts and guide targeted mitigation strategies.

**Graphical Abstract:**

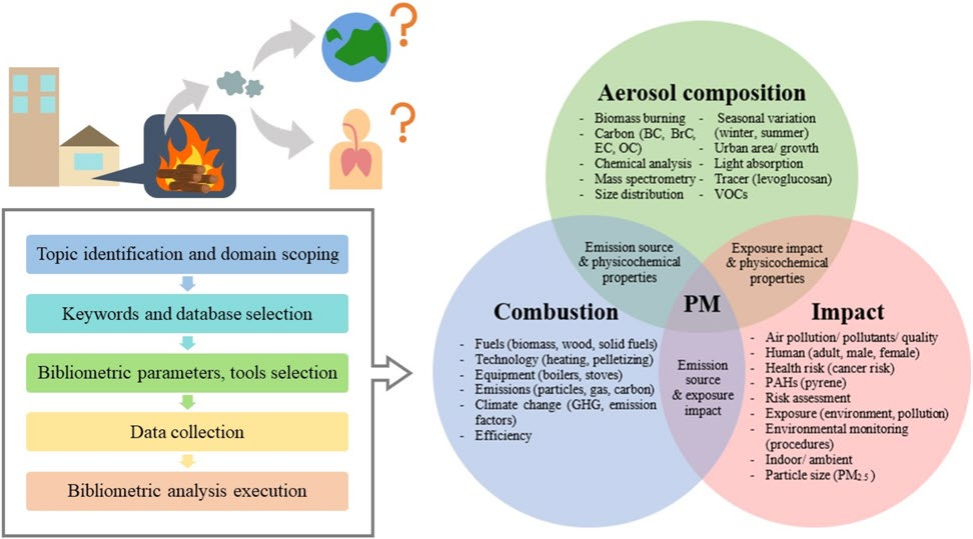

**Supplementary Information:**

The online version contains supplementary material available at 10.1007/s11356-025-36736-2.

## Introduction

In order to achieve climate neutrality, multiple approaches are being intensified including energy conservation, energy efficiency, renewable energy, and others. Bioenergy holds still today the role of the main renewable energy source globally (European Commission Joint Research Centre 2019). According to the World Bioenergy Association (WBA), bioenergy had a share of approximately 71% of the total renewables in the gross final energy consumption globally in 2019, and it dominated the renewable heat production of 96% in 2020 (Global Bioenergy Statistics 2023). Among various types of biofuels, wood in its various forms is one of the main fuels being used in the heating sector and it is the oldest energy source used by humans (Lewis 1981). Residential wood combustion (RWC) contributes significantly to energy production and heating while also creating an extensive quantity of atmospheric contaminants (Cincinelli et al. 2019). Despite its significance, wood combustion–based energy presents some disadvantages due to its impact from emitting pollutants. Today it is widely recognized that the residential burning of wood in small combustion appliances represents one of the main causes of air pollution in many parts of the world (Bari et al. 2009; Singh et al. 2023; Vicente et al. 2020; Vicente and Alves 2018). Because there are such many variations in fuel types, operational equipment and conditions, variety of chemicals, and a combination of gaseous and particulate matter are present in the emission (Singh et al. 2023); it becomes quite challenging to assess the potential environmental and health effects of these emissions (Vicente et al. 2020).

Air pollution is one of the world’s major harmful causes of environmental degradation and adverse human health effects (Environment at a Glance Indicators 2023). According to the World Health Organization (WHO), ambient and household air pollution resulted in a combined effect causing 6.7 million premature deaths annually (WHO 2023). Furthermore, an estimation was shown that 61,000 annual premature deaths are regarded as caused by outdoor air pollution from residential heating by using wood and coal in Europe, and 10,000 premature deaths in North America (*Residential Heating with Wood and Coal* 2015). Evidence illustrated the possible health implications involving asthma, lung cancer, and carcinogenicity from the air pollutants associated with residential combustion (Ali et al. 2021). Specifically, with the COVID-19 pandemic, certain studies had found that particulate matter is associated with infectious diseases and concomitant deaths in different geographic location (Jankowska-Kieltyka et al. 2021; Setti et al. 2020), raising more concerns toward the impact of airborne particles. However, the current energy situation and environmental transition goals are driving toward renewable energy, in which wood and biomass have an essential role. Global policy implications have been focusing on reassessing the full potential of wood as a source of energy, as the current consumption is still lower than the existing resource potential (Lauri et al. 2014; Wood as a Source of Energy 2017). Although substituting fossil fuels with biomass do not necessarily lead to higher emissions of air pollution in medium-to-large combustion boilers, residential scale biomass combustion can be problematic due to ineffective mixing of fuel and air (Tomlin 2021; Williams et al. 2012). The full utilization of biomass combustion on a domestic scale is not a prerequisite for an energy transition, but it is one of the alternatives. Within this context, a contrast exists between the push for energy transition and the need to limit air pollution, particularly regarding particulate matter emissions. Therefore, it is important to assess the existing research on the impacts of particles from residential wood combustion (RWC) to identify key findings, gaps, and emerging trends. Understanding the evolution of research in impact of particles from RWC can help pinpoint critical areas for further study. Subsequently, they can provide scientific evidence that can drive better policy formulation, implementation, and enforcement such as including new measurement parameters to reflect current concerns (e.g., ultrafine PM) and developing new exposure threshold to address health metrics.

In recent years, the number of scientific articles in general has been strongly increasing (Bornmann et al. 2021). Studies related to wood combustion particles is therefore expected to follow the same tendency as well. Several high-quality critical reviews exist on specific aspects of wood combustion: review on health effects of wood smoke gaseous and particle emissions (Naeher et al. 2007), particles’ characteristics and associated health effects (Kocbach Bølling et al. 2009), and toxicity of different biomass combustion exposure (Sigsgaard et al. 2015). Given the complexity and interdisciplinary nature of this field, it is increasingly challenging to keep track of the rapidly growing body of research. To the best of our knowledge, a quantity mapping of the research landscape has not been addressed yet. That is how bibliometric analysis has come to our attention as it can effectively help to define what has been well studied and what needs to be further explored. Bibliometric analysis is a methodology of reviewing large numbers of scientific research with the aid of computational software to find the main research works along with their links to one another (Bellis 2009; Han et al. 2020). Due to the tremendous number of existing articles, bibliometric review has become more highlighted thanks to its clarity in illustrating the context making it more visible for scientists and scholars to identify emerging research trends and constructive patterns among collaborations (Donthu et al. 2021). Many fields of studies have applied the use of bibliometric analysis such as energy and environment (Albort-Morant et al. 2017; Svedovs et al. 2023). Some examples of developed software to ease the bibliometric review visualization are *Bibexcel*, *Biblioshiny*, *BiblioMaps*, *CiteSpace*, *CitNetExplorer*, *Gephi*, *SciMAT*, *Sci*^*2*^* Tool*, and *VOSviewer* (Donthu et al. 2021; Moral-Muñoz et al. 2020). For this research, *VOSviewer* was used because its data-importing ability was practical, and its visualizing mode suited our research’s objectives.

In this study, a visual-quantitative bibliometric analysis approach with identification of publication directions over the last two decades is adopted. Our motivation is mainly due to two main reasons: (i) there was no bibliometric review articles done on this particular subject prior to our knowledge, (ii) the subject is an integrative multidisciplinary topic between two important research domains of wood combustion and air pollution. By having this bibliometric analysis, researchers can use the quantitative-synthesized results to complement the existing literature, and identify key research clusters and emerging themes to enhance research development. Therefore, the specific questions of this research paper are as follows: what are the main axes of the existing research and their keyword connections captured from the overview map? and what are the research trends and emerging keywords in the last two decades that deserve further investigation?

## Methodology

To construct bibliometric analysis, several methods can be brought into practice. While each study has few differentiations, the general concept is still maintained to be similar. Table 1 presents an example of the summarize methodology performed in selected recent articles.

**Table 1:** Summary of bibliometric analysis methodology from exemplary articles

Title	Year	Field of study	Summarize methodology	Ref.
Bibliometric analysis of the alternative biomass types and biomass combustion technologies	2023	Environment, biomass, combustion technologies	1. Research topic identification	Svedovs et al. (2023)
			2. Database selection	
			3. Query definition	
			4. Bibliometric parameters selection	
			5. Analysis tool selection	
			6. Collected data visualization	
How to conduct a bibliometric analysis: an overview and guidelines	2021	Business	1. Define the aims and scope of the bibliometric study	Donthu et al. (2021)
			2. Choose the techniques for the bibliometric analysis	
			3. Collect the data for bibliometric analysis	
			4. Run the bibliometric analysis and report the findings (Performance analysis, Science mapping)	
Bibliometric analysis of trends in biomass for bioenergy research	2020	Bioenergy, biomass	1. Data collection using query string in database advanced search	Ferrari et al. (2020)
			2. Data processing through cleaning, tokenization, word-sens disambiguation, stemming, and ranking	
			3. Temporal evolution and cluster analysis	

Since there are no definitive methodology to perform bibliometric study, we analyzed existing literature and studied their different steps. For the steps that have similar objectives, we grouped them together. Then, we identified four major steps: topic definition, technique selection (keywords, database, tools, parameters), data collection, and visualization. As this study includes two-merging domains, i.e., wood combustion and air pollution, we separated the keywords and database selection from other bibliometric parameters to further refine the keywords used in details. The final methodology follows consists of five key steps: (i) Topic identification and domain scoping, (ii) Keywords and database selection, (iii) Bibliometric parameters, and visualizing tool selection, (iv) Data collection, and (v) Bibliometric analysis execution.

### Topic identification and domain scoping

**Fig. 1:**
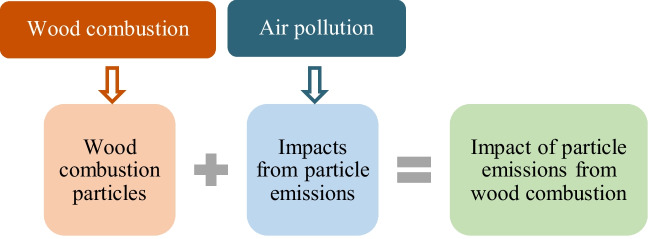
Graphical topic identification

The focus of this study is to conduct a bibliometric analysis of research on the health and environmental impacts of particle emissions from residential wood combustion. Specifically, the study examines particles originating from wood combustion, excluding particles that do not enter ambient air, such as coarse ash or those deposited in flue gas passages. To define the study scope, the topic was divided into two interconnected parts: (i) wood combustion, focusing on particle emissions, and (ii) air pollution, emphasizing the impacts of these particles, as illustrated in Fig. 1. This division was based on the interdisciplinary nature of the subject, as it spans both energy and environmental research domains. While wood combustion is primarily studied in energy and combustion science, its impacts on air pollution and public health are addressed in environmental and health studies. By structuring the analysis around these two areas, this study bridges the gap between combustion sources and particles impacts, helping to identify key research trends and areas requiring further investigation.

### Keywords and database selection

**Fig. 2:**
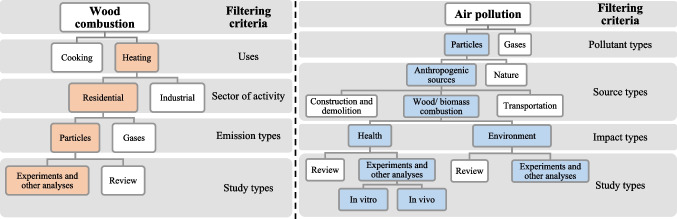
Keywords with filtering criteria. Note: The colored boxes signify focus points of the study

Keywords define the research topic and its scope (Su and Lee 2010). They help to find articles through indexing systems. In the scope of bibliometric analysis, researchers must choose a small number of keywords to reflect the domain’s essential research themes and to be able to uncover the specifics of a research core along with the connections between them at the micro-level (Chen and Xiao 2016). To capture the relevant literature, a set of keywords has to be selected. The filtering criteria established in Fig. 2 break down and highlight the study’s focus points from the existing literature, as shown in the colored boxes.

From Fig. 2, some important keywords that can help scoping down the topic are wood combustion, heating, residential, particles, and impact. As this study aims to examine both health and environmental impact types, the search did not specify a particular impact category. This approach prevents over-direction and allows the database to return all existing results, enabling the identification of key hotspots later on. Review studies are excluded to prevent keyword frequency inflation, as this study aims to visualize research trends and identify gaps based on original research (experiments, observations, data analyses, and etc.).

Three main data sources for bibliographic study are *Scopus*, *Web of Science*, and *Google scholar* (Visser et al. 2021). To avoid duplication and incomparability from different metrics of the exported data, we chose a sole database and not merging databases. *Google Scholar* was found to lack quality control needed for bibliometric analysis, because some items were not directly comparable as other databases (Aguillo 2012). For this study, *Scopus* was selected as it was found to have higher number of documents per year and covers larger number of publications compared to *Web of Science* (Visser et al. 2021). In addition, *Scopus* provides an option to download and export data in RIS and CSV formats up to 20,000 records which suits the subsequent stages of our study.

After gathering important keywords and selecting database, the proper search string must be developed as searching is the main step to achieve ethical work (Grewal et al. 2016). *Scopus* will look through the database with the given search string in the title, abstract, author keywords, and indexed keywords. Besides keywords, Boolean operators, such as AND, OR, and NOT, are necessary to enhance the findings in search engines like *Scopus*. Readers can consult (Grewal et al. 2016) for more details about the literature search, and Boolean operators’ explanation.

Table 2 presents the number of documents in *Scopus* database from different search strings. We iteratively adjusted the keywords by evaluating their coverage range and selecting those with the broadest scope when referring to similar meanings. Keywords such as particulate matters and fine particles were added to help narrow down the search by reducing large number of documents on particles, as the keyword is used in several domains. It is crucial to emphasize that these keywords are not meant to be restrictive but rather to guide the search engine in retrieving relevant documents. For example, the final set of keywords (highlighted in green box) contains residential as it covers larger number of studies compared to domestic and small-scale. However, documents that use domestic or small-scale are not eliminated as shown in the results afterwards.

**Table 2:** Number of documents per search string in *Scopus* at the time of retrieval and study parameters

Domain	Search string	No. of documents in *Scopus* database
Wood combustion particles	“wood” AND “combustion”	121,270
	“wood” AND “combustion” AND “particles”	54,158
Impact from particles	“particles”	5,168,197
	“particles” OR “particulate AND matters” OR “fine AND particles”	1,699,434
	“particles” OR “particulate AND matters” OR “fine AND particles” AND “impact”	480,705
Impact of particles from wood combustion from residential heating	“wood” AND “combustion” AND “particles” OR “particulate AND matters” OR “fine AND particles”	30,779
	“wood” AND “combustion” AND “particles” OR “particulate AND matters” OR “fine AND particles” AND “impact”	20,386
	**“wood” AND “combustion” AND “particles” OR “particulate AND matters” OR “fine AND particles” AND “impact” AND “residential” AND “heating”**	2,649
	“wood” AND “combustion” AND “particles” OR “particulate AND matters” OR “fine AND particles” AND “impact” AND “domestic” AND “heating”	1,887
	“wood” AND “combustion” AND “particles” OR “particulate AND matters” OR “fine AND particles” AND “impact” AND “small-scale” AND “heating”	1,165

The bold ones represent the final set of keywords used in this study

### Bibliometric parameters, and visualizing tool selection

The bibliometric parameters considered in this study are as follows:

- Year range: 2000–2023 (To focus on the period of significant research growth, year 2000 was set as a starting point. Documents from 1981 to 1999 accounted for approximately 2% were excluded.)
- Document type: All types of publications
- Country/territory: worldwide
- Language: All languages with at least a title and/or an abstract in English
- Data exported: citation information (author(s), document title, year, EID, source title, volume, issues, pages, citation count, source and document type, publication stage, DOI, open access), bibliographical information (affiliations, serial identifiers, PubMed ID, publisher, editor(s), language of original document, correspondence address, abbreviated source title), abstract and keywords (abstract, author keywords, indexed keywords), and references

For data visualization, *VOSviewer* developed by the Centre for Science and Technology Studies (CWTS) at Leiden University (The Netherlands) was selected thanks to the ability in its noble visualization and practicability to import the data to the software (Moral-Muñoz et al. 2020). The software is widely used as it is free and does not require external programming to perform the analysis. The limitation of *VOSviewer* is less customizable graphic results than other programming tools. We also completed a run-through on *VOSviewer* to ensure the quality and suitability with our study goals before performing the bibliometric analysis. Not only is it able to manage the selected keywords straightforwardly, but it also provides three options for visualizing the data including network, overlay, and density that we found corresponding to our objective of creating an overview map for the subject.

### Data collection

**Fig. 3:**
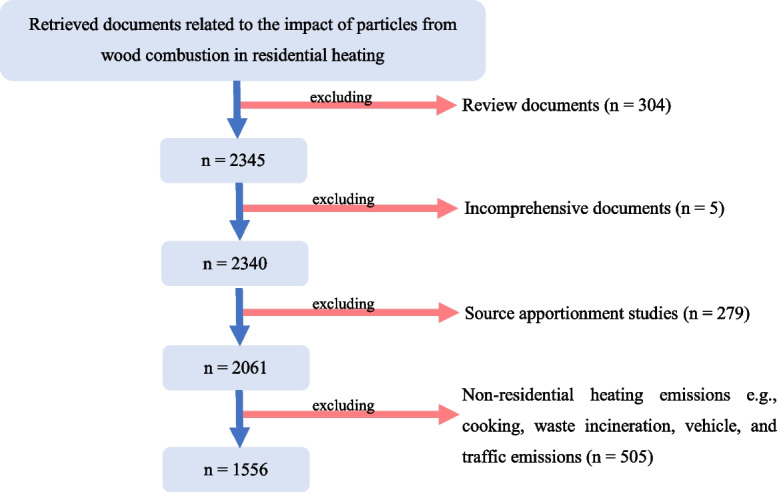
Flow chart of document screening. Note: “*n*” represents the number of documents

To this end, all documents found in the database at the search time from the following search engines were retrieved for the analysis: “wood” AND “combustion” AND “particles” OR “particulate AND matters” OR “fine AND particles” AND “impact” AND “residential” AND “heating.” The search string gave a total of 2649 documents. Despite the keywords used, some documents were not in the focus point of the study as mentioned in the “Keywords and database selection” section. For this reason, we conducted the refinement by ensuring the alignment of the retrieved results with keywords’ filtering criteria. Unrelated documents were then filtered out by screening in the search engine from titles and abstracts as illustrated in Fig. 3. It is noteworthy to mention that the screening process might not eliminate all the undesired studies, but it eases to have a narrower group of document result.

### Bibliometric analysis execution

After importing the retrieved data in *VOSviewer*, the analysis was separated into two parts containing performance analysis, and science mapping analysis. The two analyses are the two main techniques of bibliometric analysis (Noyons et al. 1999). Performance analysis evaluates research productivity by analyzing publication counts, citations, and geographical distribution to highlight influential works and trends, while science mapping applies network visualization (e.g., co-authorship, co-citation, and keyword co-occurrence analysis) to reveal relationships and evolution among keywords, researchers, and institutions.

In this study, the science mapping analysis focused mainly on two types of analysis which are in co-authorship analysis, and occurrence analysis. The purpose of the co-authorship analysis is to identify collaboration networks within the research domain. It helps to highlight connections between researchers, institutions, and countries that contribute significantly to the field, which can facilitate scientific networking and further research advancements. The co-occurrence analysis gives the ability to count the co-occurring keywords within documents (Zhou et al. 2022). It gives the information about the relationships among keywords in the form of network. Furthermore, the maps presented in the results’ section were created by using network and overlay visualization of *VOSviewer*. The density visualization was not implemented as we found that network and overlay visualization gave more clear and sufficient illustration compared to it.

In a co-occurrence network, the visualized links were created based on the number of publications in which two or more terms occur together. In this study, the authors applied the rule to limit the results to show only keywords with at least 30 occurrences from 1556 documents related to the impact of particles from wood combustion in residential heating. The reason behind applying the threshold limit is to construct a readable and informative network. In addition, some of the keywords that were almost duplicating or similarly belonged to the same meaning were removed manually to leave only one representative by the authors. For example, polycyclic aromatic hydrocarbon was selected to be the representative keyword of polycyclic aromatic hydrocarbon, polycyclic aromatic hydrocarbons, PAH, and PAHs.

## Results and discussion

### Performance analysis results

#### Publications over time: 2000–2023

**Fig. 4:**
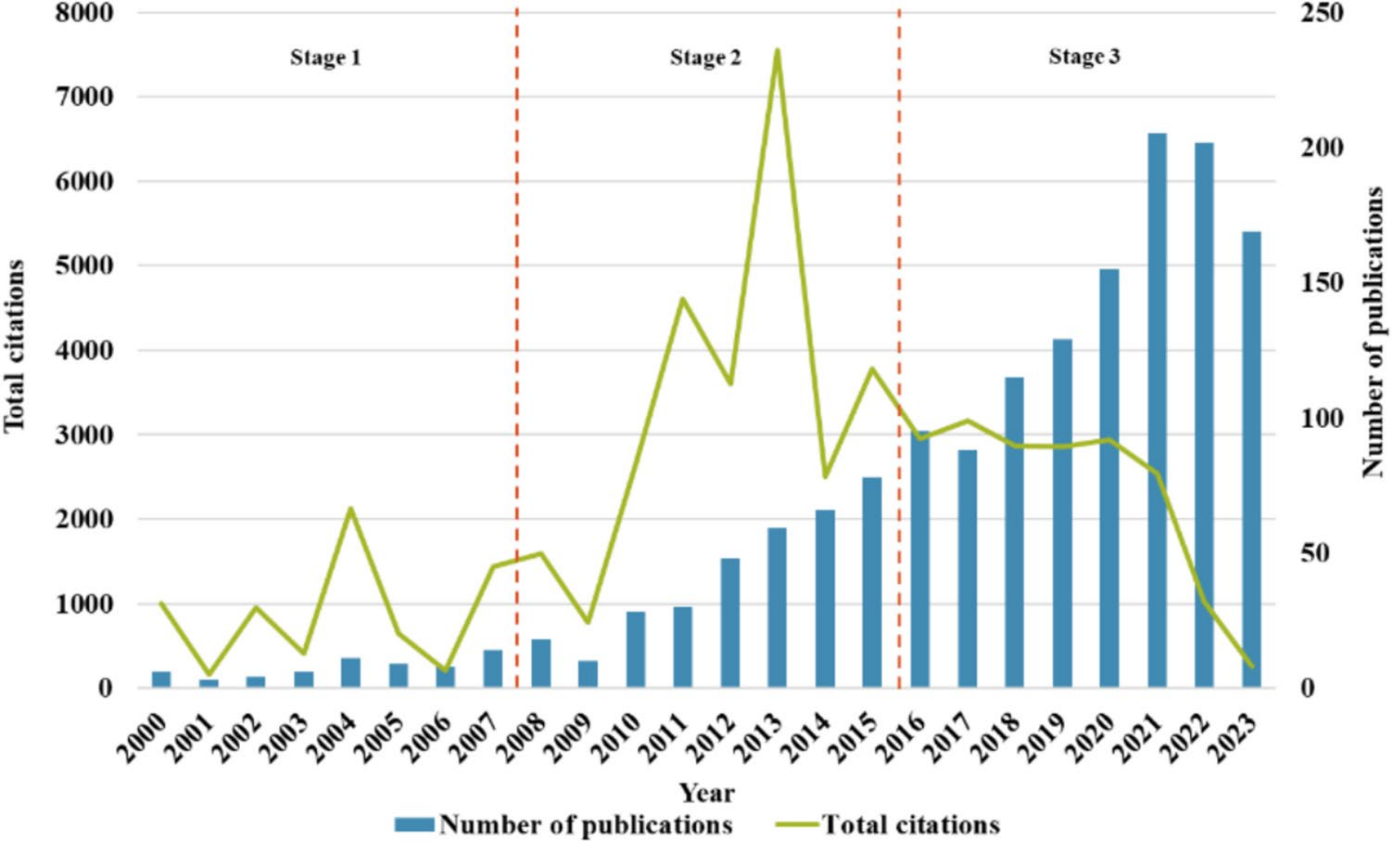
Annual total citations and number of publications related to the impact of particles from wood combustion in residential heating and over 2000–2023

In general, the research growth in most of the topics tends to increase over time. Nevertheless, the growth rate of each topic can be different due to the significance of the topic during dedicated timing. To observe the growth of the study in the theme of the impact of particles from wood combustion in residential heating, 1556 documents were selected from the methodology mentioned earlier, and they were investigated over time. It can be seen that the number of publications increases over time as in Fig. 4. The significant increases in the number of publications were similarly observed for air pollution research (Dhital and Rupakheti 2019), renewable energy studies (Guchhait and Sarkar 2023), and climate change research which was specifically found to be doubled every 5 to 6 years (Haunschild et al. 2016). In this study, the elevation of the number of documents was distinguished in three stages to better concentrate on the evolvement. To clarify, the period of 2000–2023 was equally divided into three consecutive stages of 8-year duration each as follows: stage 1 (from 2000 to 2007), stage 2 (from 2008 to 2015), and stage 3 (from 2016 to 2023).

Stage 1 is the starting phase of the study in which the average number of documents per year is 8. In stage 2, the number of documents grew rapidly with an average of 42 documents published per year. An observation was made that the growth in the number of studies in stage 2 could result from the augmentation in renewable energy development after the world economic crisis in 2008, intensive climate change concerns during COP15 in 2009 (*Green Stimulus after the 2008 Crisis–Analysis* 2020), and increased European level funding to investigate biomass combustion emissions and to develop clean technologies for biomass combustion systems such as European Commission focus on Clean Air Technology for Biomass Combustion Systems (BioCAT) in 2011 (*Clean Air Technology for Biomass Combustion Systems (BioCAT) | BIOCAT Project | Fact Sheet | FP7* 2017). In addition, ERA-NET Bioenergy programme had funded several large European projects on this topic (see (*ERA-NET Bionergy | ERA-NET BIOENERGY | Project | Fact Sheet | FP6* 2013; Tissari 2013)). Along with the risen implementation of the EU Ecodesign directive (Directive 2009/125/EC) (The European Commission 2009), emissions from energy-using products were being concentrated. More importantly, the number of documents related to the focus topic is recognized to have an important increase in stage 3 with an average of 145 documents published annually. The cause of the increase could be a wave from the war on pollution in China in 2014 (Greenstone et al. 2021), New Source Performance Standards by the U.S. EPA in 2015 (US EPA 2016), the Paris Agreement in 2015 (Paris Agreement 2015), and the implementation of Low Emission Zone (LEZ) (Gu et al. 2022). The results signify the fact that much attention has been paid to this domain up to recently. Despite the peak in the last few years, possibly due to the COVID-19 situation creating an overall increase in publication (Rousseau et al. 2023), the study related to the impact of particles from wood combustion in residential heating is expected to grow in the upcoming decades.

For citation counts, it is common to have a decrease at the end of the study period as it will be further raised and updated in the database as cited by the future publications. However, a considerable number of total citations was found in 2013 implying that there must be crucial information established and being used from the studies in that period. The peak was found to be from a study associated with the role of black carbon (BC) in the climate system by Bond, T.C., et al., which has been cited 4233 times and gained the 99^th^ percentile of *Scopus* citation. The key finding is the estimation result that BC is the second most significant anthropogenic emission after carbon dioxide for its +1.1 W/m^2^ total climate-forcing (Bond et al. 2013). The study potentially creates a strong interest in assessing BC’s impact on the climate system and prioritizing mitigation measures. In addition, the study comprehensively combines immense findings from different assessments, pollution sources, and modeling, making it a comprehensive of information.

#### Publications by geographic distribution

**Fig. 5:**
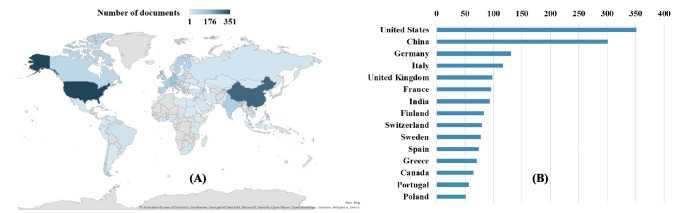
**A** Global distribution of documents related to the impact of particles from wood combustion in residential heating during 2000–2023. **B** Top 15 countries in the total number of documents related to the impact of particles from wood combustion in residential heating during 2000–2023

To analyze the contribution of different countries, countries based on authors’ affiliations are taken into consideration to ensures that all involvement receive recognition. The global distribution of documents related to the impact of particle emissions from wood combustion in residential heating was studied and is presented in Fig. 5A. The top 15 countries are shown in Fig. 5B for better illustration.

As presented, the USA published the most documents with a total of 351 documents over 2000–2023, followed by China, and Germany with a quantity of 301, and 131 documents respectively. Some other countries that have mainly contributed to the domain are mostly from Europe. Leading countries in publication numbers are potentially due to their strong environmental policies which subsequently influence funding availabilities for scientific research and infrastructure. Evident examples for strong environmental policies are U.S. EPA standards for the USA, and EU directives on air quality for European countries. This creates a demand for scientific data as well as an increase in research and development in industrial sector. To illustrate, examples of world-leading manufacturer in particles measurement equipment are Palas GmbH from Germany and Dekati Ltd. from Finland. Looking into Asian countries, India also played a significant part aside from China, as it was ranked the seventh place with 94 documents. China and India rapid growth in urbanization and industrialization could result in the research increase to minimize severe air pollution issues, as millions of premature deaths occurred annually in both countries (Khilnani and Tiwari 2018; Zhong et al. 2019). The underrepresentation of certain regions (such as parts of Africa, Asia, and South America) could possibly be due to limited support systems and infrastructure. This may result in publishing challenges, including limited access to data collection, and language barriers. It is important to note that the geographic distribution result does not alone represent the awareness and interest in the subject of each country. Yet, this is an illustration of the integrated factors including two possible elements mentioned previously, accessibility, available technology and equipment (e.g., measurement instruments, analyzers), performance, number of researchers and institutions, and others related to performing wood combustion particle emission studies. Some countries with severe RWC pollution emissions may lack the capacity to conduct research effectively, which highlights the need for global research collaboration and capacity-building initiatives.

### Science mapping results

#### Co-authorship analysis

**Fig. 6:**
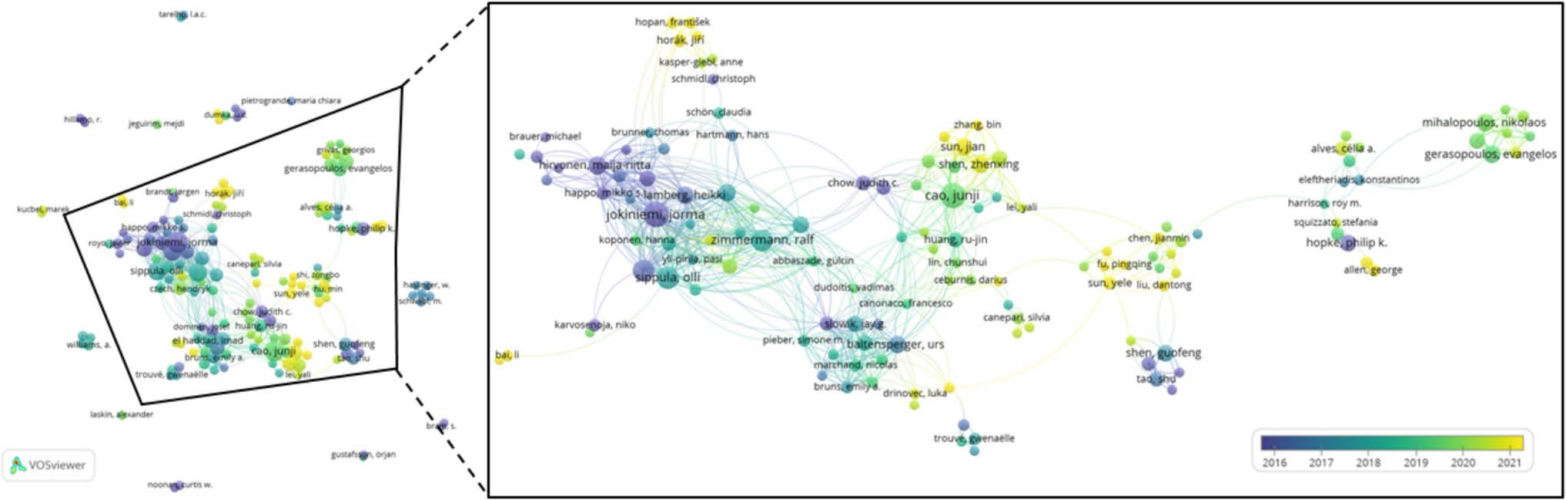
Co-authorship overlay visualization for documents related to the impact of particles from wood combustion in residential heating

For the co-authorship analysis, the restriction was made to present only the names of authors who have published a minimum number of five documents. This criterion was set to illustrate authors contributing consistently to the knowledge. As a consequence, Fig. 6 represents the co-authorship overlay visualization for documents related to the impact of particles from wood combustion in residential heating. The timeline from 2016 to 2021 was specified to better present the visible changes over time. The colors indicate the average publication year. In addition, the different size of circles reflects the number of publications from each author. Out of the 160 results, 130 items were interconnected through the main co-authorship network. Therefore, we expanded these 130 items for detailed visualization, as shown in Fig. 6.

**Fig. 7:**
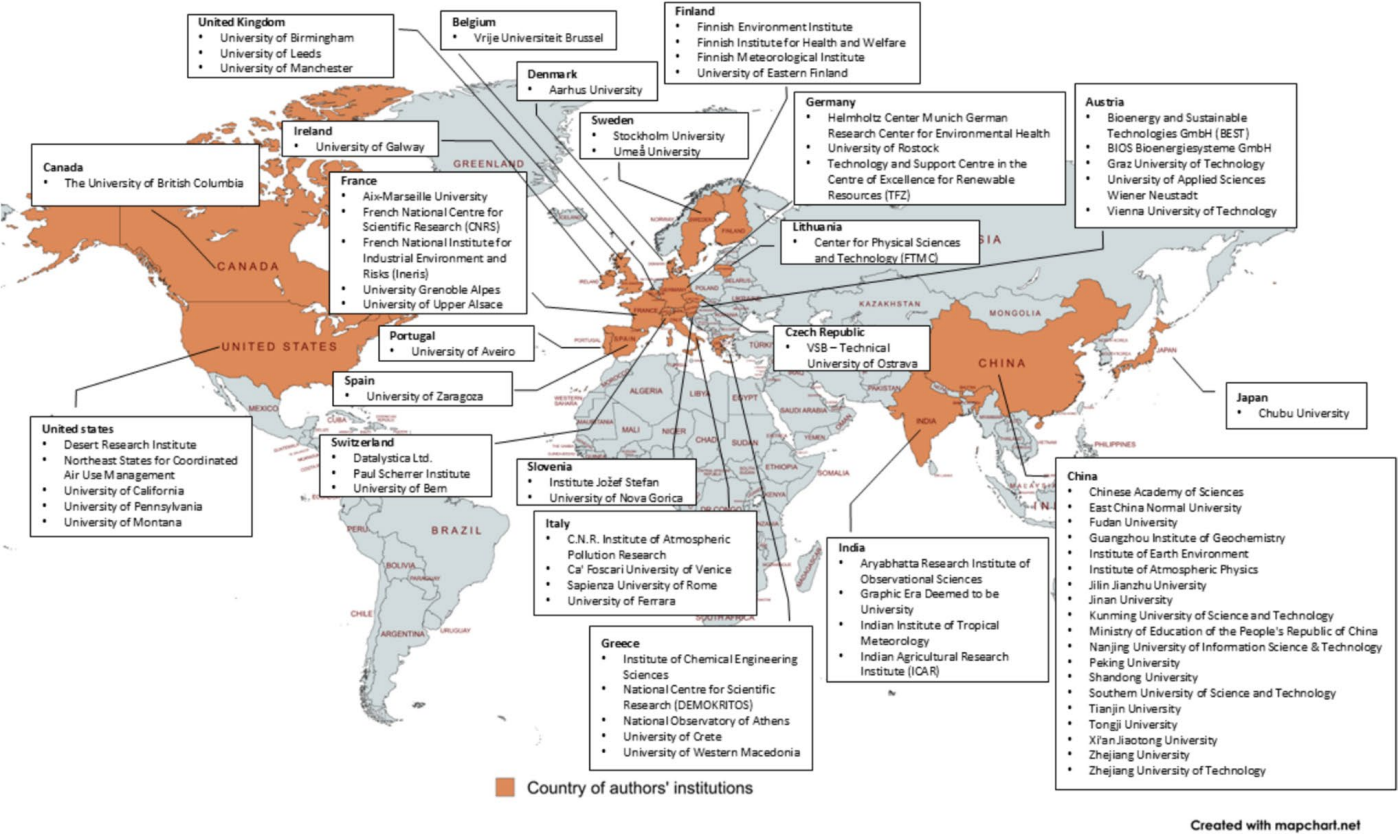
Geographic distribution of author-affiliated institutions from the co-authorship analysis

From the obtained results, we gathered the institution names of all 160 authors from Scopus and presented in Fig. 7. The co-authorship network includes researchers from nearly 80 institutions across 22 countries, highlighting the wide geographic and institutional spread of collaboration. The full list of author names and institutions (retrieved from *Scopus*) can be found in Table S1. This information gives the possibility for researchers to discover, collaborate, and deepen upcoming research work within the specialization. The limitation of this analysis is the author’s names used in each publication, as they might be written differently making it difficult to be recognized and counted by the software.

Among the 160 authors identified in the co-authorship analysis, Table 3 highlights the top 25 based on total link strength. We selected the top 25 authors because beyond this number, several authors share equal total link strength values. The total link strength reflects how many times the researcher has co-authored with each collaborator. This reveals key authors who are central nodes within the co-authorship network. As these authors have high levels of collaborative activity, their role in fostering academic connectivity within the field is significant. A high total link strength reflects not only frequent collaboration but also a wide network of co-authors. This may result from sustained productivity within research group as well as active participation in multi-institutional or international collaborations. It should be pointed out that all authors aside from the presented results in this study are likewise meaningful to the enlargement of knowledge of the subject.

**Table 3:** Top 25 authors by total link strength in co-authorship analysis

Author names	Institution	Total link strength	No. of documents (from 1556 documents)
Jorma JOKINIEMI	University of Eastern Finland	184	30
Olli SIPPULA	University of Eastern Finland	137	23
Ralf ZIMMERMANN	Helmholtz Center Munich German Research Center for Environmental Health, University of Rostock	126	22
Jarkko TISSARI	University of Eastern Finland	125	22
Junji CAO	Institute of Atmospheric Physics, Chinese Academy of Sciences	107	28
Maija-Riitta HIRVONEN	University of Eastern Finland	107	16
Heikki LAMBERG	University of Eastern Finland	94	14
Pasi I. JALAVA	University of Eastern Finland	85	12
Miika KORTELAINEN	University of Eastern Finland	85	10
Hendryk CZECH	Helmholtz Center Munich German Research Center for Environmental Health	84	11
Jay Gates SLOWIK	Paul Scherrer Institute	81	12
Jürgen ORASCHE	Helmholtz Center Munich German Research Center for Environmental Health	77	14
Imad EL HADDAD	Paul Scherrer Institute	69	12
André S.H. PRÉVÔT	Paul Scherrer Institute	68	10
Urs BALTENSPERGER	Paul Scherrer Institute	66	15
Oskari USKI	Umeå University	65	9
Zhenxing SHEN	Xi'an Jiaotong University, School of Basic Medical Sciences	64	15
Mikko S. HAPPO	University of Eastern Finland	62	9
Jian SUN	Xi'an Jiaotong University	61	14
Jürgen SCHNELLE-KREIS	Helmholtz Center Munich German Research Center for Environmental Health	61	13
Nicolas MARCHAND	Aix-Marseille University, French National Centre for Scientific Research (CNRS)	58	8
Emily Anne BRUNS	Paul Scherrer Institute	55	8
Giulia STEFENELLI	Paul Scherrer Institute	55	7
Simone M. PIEBER	University of California	53	7
Amelie BERTRAND	Paul Scherrer Institute	51	7

#### Co-occurrence analysis

**Fig. 8:**
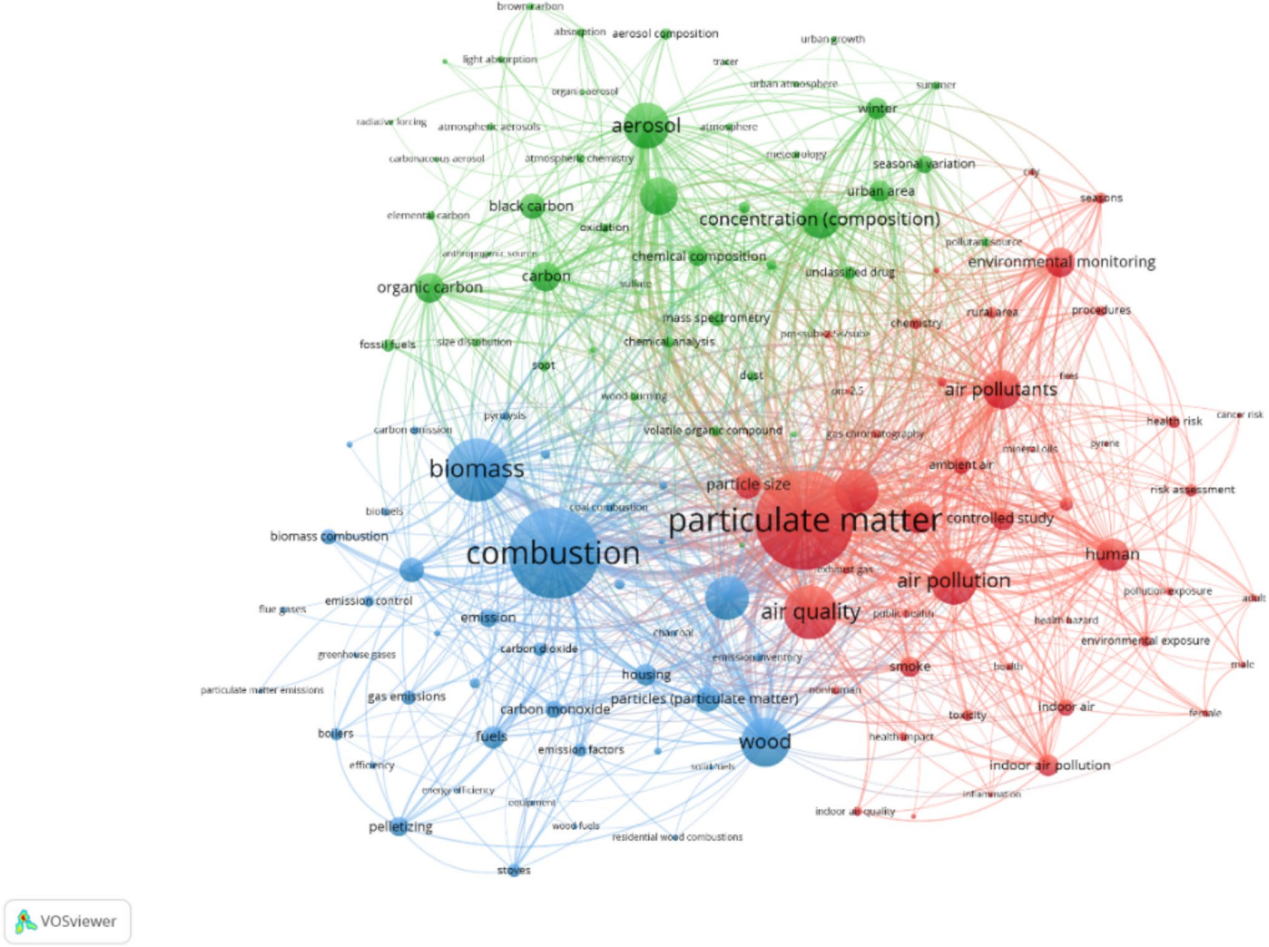
Co-occurrence network visualization with the minimum number of 30 occurrences per keyword for documents related to the impact of particles from wood combustion in residential heating

**Fig. 9:**
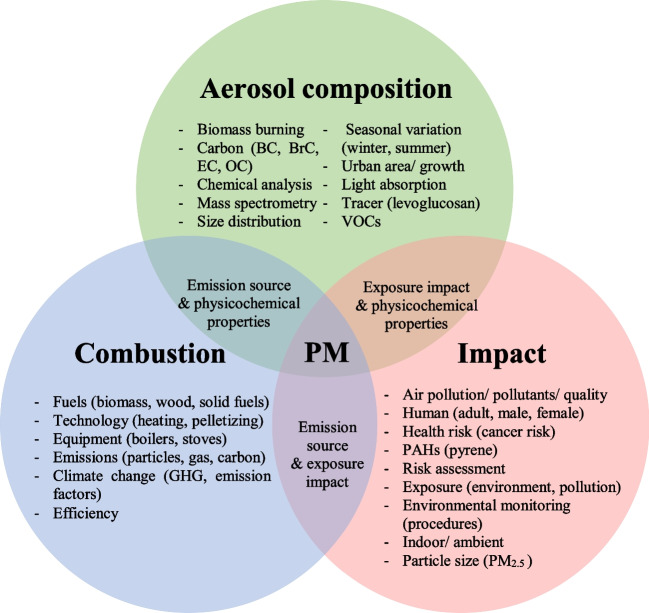
Venn diagram of co-occurrence keywords

**Fig. 10:**
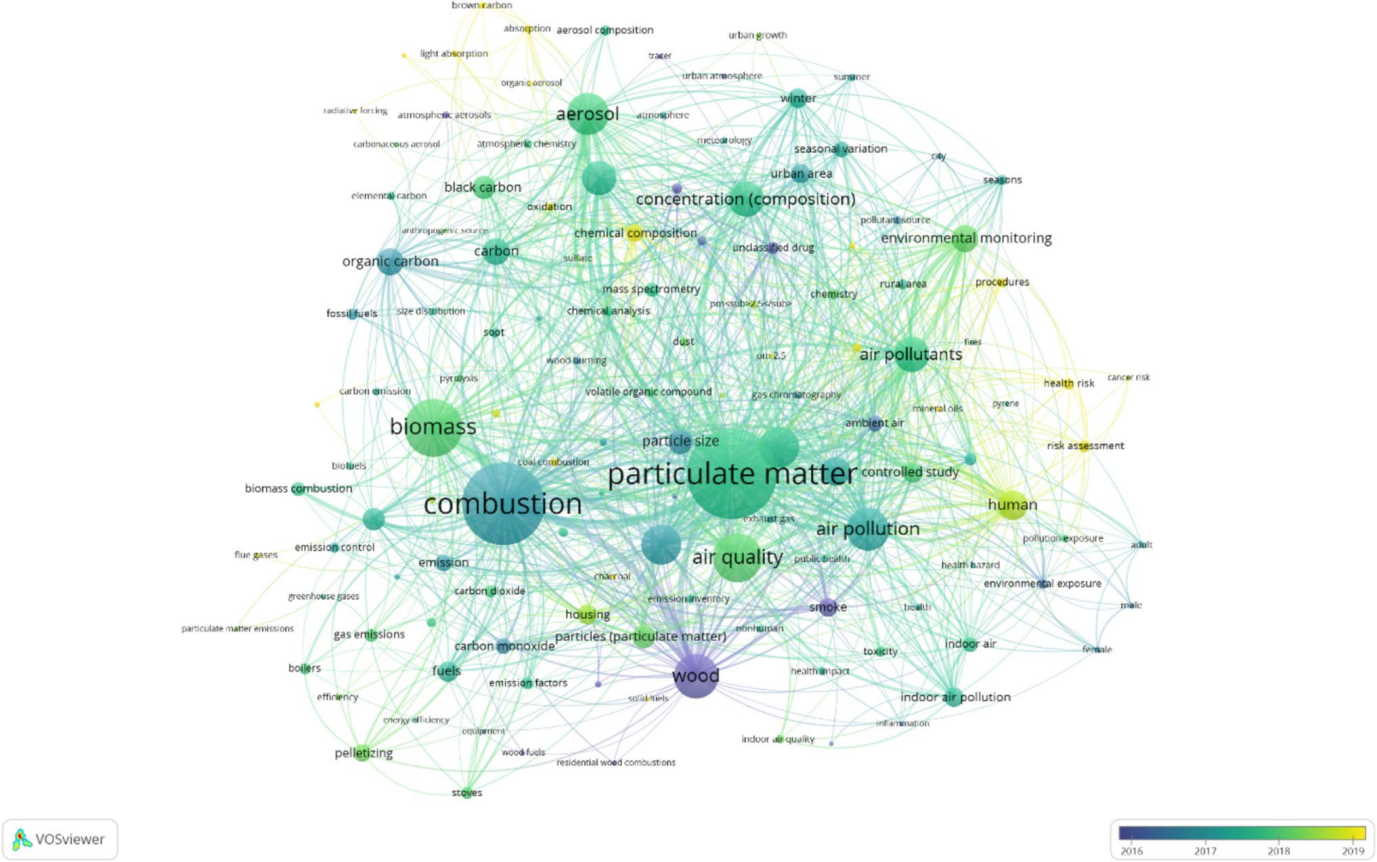
Co-occurrence overlay visualization with the minimum number of 30 occurrences per keyword for documents related to the impact of particles from wood combustion in residential heating

After the keywords have been cleaned and treated, the network visualization of the co-occurrence analysis from 1556 documents gave the result as shown in Figs. 8 and 10. The circle size represents the number of documents for each keyword. In Fig. 8, the keywords were divided into three clusters as represented in different colors. The main keywords were then organized in group as shown in Venn diagram in Fig. 9 to ease the conception.

The blue cluster is mostly highlighting the keywords linked to the combustion domain. It showed the interconnection between important combustion topics such as fuels (biomass, and wood), technology (heating, pelletizing), and equipment (boilers, and stoves). It can also be considered as an encouragement toward climate change mitigation as the keywords such as greenhouse gases and emission factors are linked and recognizable to the combustion aspect in this cluster.

For the green cluster, the main concepts gained from the picture are aerosol, biomass burning, and concentration (composition). The visualization gave a great number of keywords related to important particulate constituents that should be considered in the research in this particular domain. When biomass is burning, it is usual to take into consideration various types of carbon classification including black, brown, elemental, and organic carbon. Besides, a commonly used chemical tracer that can be used in tracking biomass burning emissions is levoglucosan. The keywords associated with seasonal variation (winter, summer), and town-use (urban area, urban growth) are also intriguing to be seen in this cluster, emphasizing the influence on the emissions’ compositions.

The red cluster explicitly shows the keyword of particulate matter. In this cluster, the domain of impact from particles stood out as it contains other major keywords, e.g., human, health risks, toxicity, and environmental exposure. The links between particulate matter and health or environmental impact are not that strong and well-established compared to the links between particulate matter and atmospheric pollution. This could be due to current regulations such as WHO air quality guidelines (WHO 2021) and EU Air Pollution Directive (The European Commission 2008) that focus primarily on PM mass concentration rather than PM composition and toxicity. Underlying this gap as a signal to enhance environmental impact and health effect studies may lead to a better understanding of pollution awareness. Some health implications showed up among the other possibilities, for instance, cancer risk and inflammation. Identifying hazardous PM impacts can guide policy decisions on representative emission limit.

The three important keyword clusters, namely aerosol composition, combustion, and impact as presented in Fig. 9, can be an effective suggestion for further research work to explore the related studies. By using these guided keywords, future researchers can obtain an overall existing knowledge to conduct the research involving impacts from residential wood combustion.

Another aspect that was obtained thanks to VOSviewer visualization is an overlay map. Overlay visualization presented in Fig. 10 is an illustration of co-occurrence analysis as in Fig. 8 but the difference lies in the color separation. Here, the color of each keyword was defined by its average time of publication. Therefore, overlay visualization can be extremely useful in determining research evolution and identifying emerging research areas. As the main changes existed from 2016 to 2019, this period was selected to highlight the significant emerging trend and to observe the visible change from the entire period of 2000 to 2023.

The studies related to wood and smoke were published averagely before and during 2016. Then, it was transferred through links between other keywords a year or a few years later. On the other hand, keywords associated with humans began to be visible in the period between 2018 and 2019. It emphasizes the potential fact that the human health-related theme was explored mostly from the indicated timing. This may be attributed to the awareness raised in major events including the first global conference on air pollution and health organized by WHO in 2018 (WHO 2018), amended Gothenburg protocol entering into force in 2019 setting emission reductions commitments specifically for PM_2.5_ and targeting pollutants like black carbon (CEIP 2019), and the aftermath of COVID-19 pandemic. It is important to remark that the keyword “human” is quite far from the keyword “combustion.” This represents that they have probably co-used and co-occurred but they are not strongly related. Besides, there is a lack of link between “health” and “chemical composition” in which both play a significant role in determining impact on human exposure, whereas the keyword “particulate matter” has a better interlink to the keywords “human” and human-related theme. Further observation was made that there is no presence of keywords regarding secondary organic aerosols (SOA) signifying the necessity in more investigation in this sector. Certain publications regarding SOA gathered in this research were focusing on precursors (Bruns et al. 2016; Růžičková et al. 2022), mitigation (Pieber et al. 2018), ageing of emissions, and their contribution to mortality (Nault et al. 2021).

On the upper part of the figure, there are other emerging keywords for 2019 which are chemical composition, light absorption, brown carbon, and organic aerosol. This could also be used in investigating different study themes related to environmental impact as some of these keywords play a key role in defining pollutants’ effect on global climate such as light absorption. Nevertheless, a few supplemental keywords explored in 2019 were scattered over the figure but did not show a significant relation in a group form so they were not considered in the result analysis of a current study.

**Fig. 11:**
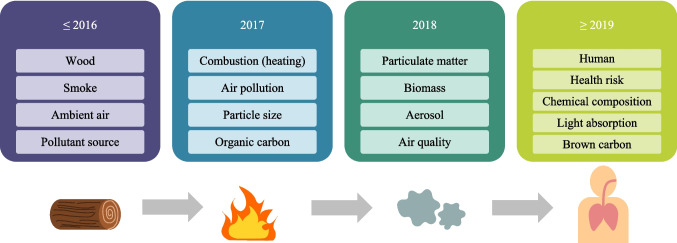
Co-occurrence keywords evolution

To perceive the result presented in the overlay visualization, Fig. 11 illustrates the evolution of co-occurrence keywords in the period with the highest visible change. The main keywords for each year were selected and illustrated.

### Research trend and emerging direction

This section provides further information on research trends gathered from the bibliometric analysis performed in this study. Furthermore, recent literatures, notably from 2018 to 2023, were pulled to comprehend the analysis. According to the results described in the “Co-occurrence analysis” section, we were capable of investigating the evolution of the topic and identifying three main groups of keywords that were introduced in recent years. Hence, we underscored the following keywords and conducted a more detailed review for each keyword associated with the impacts of particles from wood combustion in residential heating.

#### Human health–related risk and risk assessment

Emissions caused by residential wood combustion are major contributors to indoor and outdoor PM pollution impacting human health (Olsen et al. 2020). Evidence has shown that increasing exposure to PM_2.5_ which belongs to airborne fine particles can lead to a decrease in life expectancy (Martins and Carrilho Da Graça 2023). A life expectancy could be shortened by 1–1.6 years for using the fireplace in a living room for 4 h per day in the evening while using woodstoves in the same period for a single-family townhouse building could reduce to a cumulative DLE of half a year (Martins and Carrilho Da Graça 2023). More specifically, biomass-fed heating systems emit a crucial number of particles resulting in high exposure and excess cancer risk (Stabile et al. 2018). Both studies agreed that open fireplaces cause a higher risk of damaging human health than closed wood-burning equipment or woodstoves with external air supply (drawing outdoor air for combustion). Further study done on lung-deposited doses of PM exposure also suggested upgrading the wood-burning technology to reduce PM levels, as it was found that using a fireplace led to a twofold higher total dose of PM received than a woodstove (Vicente et al. 2021). Besides impacts from indoor exposure, PM from residential combustion wood smoke also effects human health via outdoor exposure (Naeher et al. 2007). The contributing PM mass in ambient air depends on seasonal variation, source density, employed technology, and location (Sigsgaard et al. 2015), most of which were also reflected in the co-occurrence keywords of this study. Evidence showed the existing influence of the physicochemical properties on biological effects of PM (Kocbach Bølling et al. 2009). Consistent with previous study, our research also captured key links, not only between chemical properties but also between particle size and health-related metrics. Studies found that PM_2.5_ from RWC induced multi-cytotoxicity via upregulating oxidative stress (Sun et al. 2023) and lifetime cancer risks from inhaling PM_2.5_ bound PAHs (Alves et al. 2023), while another toxicological study focusing on smaller size as PM1 due to their penetration ability (Jalava et al. 2010) highlighted that smouldering PM caused higher inflammation than normal PM. Thus, we emphasize the need for integrative assessment frameworks of PM impacts in relation to their physicochemical properties, notably different size distribution and chemical characteristics, to reflect the health risks of exposing RWC particles for both freshly emitted and outdoor aging particles. Future research could aim at developing emission factor associating with health risk factor in parallel.

#### Brown carbon and light absorption

Carbonaceous particles are usually emitted from different sources of combustion, especially biogenic sources including wood and biomass combustion, and they play a critical role in absorbing and scattering solar radiation (Haywood and Boucher 2000; Kanakidou et al. 2005; Zhang et al. 2020a, b). Carbonaceous aerosols include black carbon (BC) or elemental carbon (EC), and organic carbon (OC) (Li et al. 2023). Before, OC was believed to have only scattering ability; however, it was found that brown carbon (BrC) existing within the compounds of OC has the ability to highly absorb UV and short-visible wavelengths (Laskin et al. 2015; Liu et al. 2014; Paraskevopoulou et al. 2023). That is to say, BrC refers to a fraction of OC that absorbs light depending on the wavelengths (Kirchstetter et al. 2004; Sun et al. 2021) and is mainly emitted from residential biomass combustion (Zhang et al. 2020a, b) for anthropogenic source, and wildfires (Xiong et al. 2022) for natural sources. The fraction of solar radiation that is absorbed by BrC, relative to that of the total PM, was observed to be in a range of 0.141 to 0.609 among different combustion stages (Chen et al. 2024). Various studies have found that BrC can potentially contribute to climate change (Li et al. 2023; Tian et al. 2023) including directly affecting the climate by increasing air temperature (Drugé et al. 2022), and causing snow and/or ice albedo effect (Tuccella et al. 2021). Additionally, atmospheric aging such as oxidation, shown among BrC and chemical composition keywords, can alter light absorption properties (Kuang et al. 2021). A study found an importance in incorporating seasonal variation of BrC fractional solar absorption as it affects radiative balance and air quality model (Huang et al. 2018). To this end, future research could explore regional and global impacts of BrC by combining field studies and air modeling to gain deeper understanding and advance model development. These results could be also incorporated into emission pollution control programs concerning emissions of RWC in the long term.

#### Chemical composition

The emissions of RWC are influenced by many factors such as combustion technology, fuel types/species, and user practices (Jalava et al. 2012; Johansson et al. 2004; Orasche et al. 2013), resulting in different PM characteristics worldwide. Recent study had shown that smoldering biomass combustion released higher PAH fraction with high molecular weight than flaming period (Zhang et al. 2022). It is clearly known that altered chemical compositions will vary regarding their toxicological potential. In order to evaluate PM exposure effects, PM characteristics for example particle size, porosity, morphology, and chemical composition have to be defined (Moreno-Ríos et al. 2022). To illustrate, the toxicity of particles is different for each chemical profile from freshly emitted particles to aging particles (Sun et al. 2022). In addition, there is a growing interest in identifying and quantifying specific organic compounds emitted during wood combustion, involving polycyclic aromatic hydrocarbons (PAHs), oxygenated organic compounds, and other volatile organic compounds (VOCs) in different regions (Alves et al. 2023; Hettiyadura et al. 2021; Pietrogrande et al. 2021). Thus, a contribution toward the understanding of RWC particles’ chemical composition is essential for assessing their environmental and health impacts. This could be achieved by combining advanced analytical techniques such as investigating chemical composition and possible PM transformation with gas chromatography-mass spectrometry analysis (Bruns et al. 2017; Sei et al. 2021; Wang et al. 2023). These approaches could be performed under controlled experiments, and real-world conditions monitoring in a continuous line. As we observed in this study, many potential research collaborations can be created to connect the dots of the knowledge. By integrating the mentioned approaches, researchers can therefore accurately access the link between chemical compositions and their environmental and health impacts leading to better guidelines and mitigation strategies.

## Conclusion

This paper is a pioneer study that implements a bibliometric perspective to visually analyze published research works on RWC particles’ impacts aimed at the comprehensive understanding of knowledge in this field and to identify directives for future research. The study reviewed papers published during 2000–2023 related to our objectives. It was observed that the number of publications shows steady growth with different rates and different periods depending on energy situations and environmental concerns. It is confirmed that it is evident that wood combustion in residential heating poses significant links to human health risks and climate effects, including a decrease in life expectancy, excess cancer risk, and global temperature rise. Recent studies have highlighted the need for risk assessment frameworks to analyze the impact of particle emissions on human health and the environment, emphasizing the importance of upgrading wood-burning technology to reduce pollution levels. Additionally, the chemical composition of particulate emissions from different wood species and combustion conditions influences their toxicological potential, indicating the need for further research in this area.

The cluster analysis from co-occurrence network visualization can be used to address the first research question that three major axes of the existing research are combustion, aerosol composition analysis, and health effects from PM. Thus, our primary suggestion is to emphasize that future studies should contain these three important pillars within the study. Owing to the co-occurrence overlay visualization, the research evolvement is observed mostly from 2016 to 2019. To answer our second question, we identified three aspects that were introduced in recent years and must be developed more in the future: (i) human health-related risk assessment, (ii) brown carbon and light absorption, and (iii) chemical composition. In conclusion, our bibliometric review has given meaningful results and responded to our objectives in illustrating the overview map of research on the impacts of particles from wood combustion in residential heating. Concerns about particles from residential combustion in the heating sector have been raised for its impacts through research. Many studies have made a crucial effort to knowledge contribution on human health, but overall, the aspect is still limited. Research challenges are to identify explicit impacts such as pulmonary inflammation, carcinogenicity, and genotoxicity, along with updating representative wood types and operation conditions to depict real-life utilization. The presented results are important for the future development of wood/biomass energy in every sector because they highlight the need and urgency in implementing representative regulation to better address exposure limit, such as integrating PM size distribution, number concentration, and chemical analysis in addition to mass concentration measurement. As we observed an obvious link between PM composition from RWC combustion with health impacts, adding physicochemical parameters in a real-world scale can therefore help to assess potential risk and toxicity. Incorporating chemical and optical properties could help to enhance environmental impact understandings for long-term emission mitigation. This will lead to the development of emission reduction and control technology promptly along with policy implementation to support the sustainable use and management of wood in residential heating.

## Supplementary Information

Below is the link to the electronic supplementary material.

Supplementary file1 (DOCX 27 KB)

## Data Availability

The data supporting the findings of this study are available within the paper. Further information generated during the study are available from the corresponding author on reasonable request.

## Abbreviations

BC: Black carbon
BrC: Brown carbon
CWTS: Centre for Science and Technology Studies
DLE: Decrease in life expectancy
EC: Elemental carbon
GHG: Greenhouse gases
LEZ: Low emission zone
OC: Organic carbon
PAH(s): Polycyclic aromatic hydrocarbon(s)
PM: Particulate matters
RWC: Residential wood combustion
SOA: Secondary organic aerosols
TVOC: Total volatile organic compound
U.S. EPA: United States Environmental Protection Agency
VOC(s): Volatile organic compound(s)
WBA: World Bioenergy Association
WHO: World Health Organization
