# Supplementary material for: A comprehensive bibliometric analysis of research on health and environmental impacts of particle emissions from wood combustion in residential heating
Source: Environ Sci Pollut Res Int. 2025 Jul 17;32(30):17897–915. doi: 10.1007/s11356-025-36736-2 (PMC12328525; doi:10.1007/s11356-025-36736-2)
Supplement: Supplementary file 1 — Supplementary file1 (DOCX 27 KB) [file 11356_2025_36736_MOESM1_ESM.docx]

Table S1. List of authors and institutions in the co-authorship network.

| Author names | Research institution | Country |
| --- | --- | --- |
| Harald STRESSLER | Bioenergy and Sustainable Technologies GmbH (BEST) | Austria |
| Manual SCHWABL | Bioenergy and Sustainable Technologies GmbH (BEST) | Austria |
| Rita STURMLECHNER | Bioenergy and Sustainable Technologies GmbH (BEST) | Austria |
| Walter HASLINGER | Bioenergy and Sustainable Technologies GmbH (BEST) | Austria |
| Ingwald OBERNBERGER | BIOS Bioenergiesysteme GmbH | Austria |
| Christoph HOCHENAUER | Graz University of Technology | Austria |
| Thomas BRUNNER | Graz University of Technology | Austria |
| Christoph SCHMIDL | University of Applied Sciences Wiener Neustadt | Austria |
| Gabriel REICHERT | University of Applied Sciences Wiener Neustadt | Austria |
| Anne KASPER-GIEBL | Vienna University of Technology | Austria |
| Magdalena KISTLER | Vienna University of Technology | Austria |
| Jacques DE RUYCK | Vrije Universiteit Brussel | Belgium |
| Svend BRAM | Vrije Universiteit Brussel | Belgium |
| Michael BRAUER | The University of British Columbia | Canada |
| Shaofei KONG | China University of Geosciences | China |
| Chunshui LIN | Chinese Academy of Sciences | China |
| Jie TIAN | Chinese Academy of Sciences, Ministry of Education of the People's Republic of China | China |
| Shichang KANG | Chinese Academy of Sciences | China |
| Yali LEI | East China Normal University, Ministry of Education of the People's Republic of China | China |
| Jianmin CHEN | Fudan University | China |
| Xinming WANG | Guangzhou Institute of Geochemistry, Chinese Academy of Sciences | China |
| Junji CAO | Institute of Atmospheric Physics, Chinese Academy of Sciences | China |
| Renjian ZHANG | Institute of Atmospheric Physics, Chinese Academy of Sciences | China |
| Yele SUN | Institute of Atmospheric Physics, Chinese Academy of Sciences | China |
| Zifa WANG | Institute of Atmospheric Physics, Chinese Academy of Sciences | China |
| Haiyan NI | Institute of Earth Environment, Chinese Academy of Sciences | China |
| Jing DUAN | Institute of Earth Environment, Chinese Academy of Sciences | China |
| Qiyuan WANG | Institute of Earth Environment, Chinese Academy of Sciences | China |
| Li BAI | Jilin Jianzhu University, Ministry of Education of the People's Republic of China | China |
| Jun TAO | Jinan University | China |
| Wei DU | Kunming University of Science and Technology, Ministry of Education of the People's Republic of China | China |
| Yan-Lin ZHANG | Nanjing University of Information Science & Technology, Ministry of Education of the People's Republic of China | China |
| Guofeng SHEN | Peking University, Ministry of Education of the People's Republic of China | China |
| Min HU | Peking University, Ministry of Education of the People's Republic of China | China |
| Shu TAO | Peking University | China |
| Siye WEI | Peking University | China |
| Tong ZHU | Peking University | China |
| Liang XU | Shandong University | China |
| Huizhong SHEN | Southern University of Science and Technology | China |
| Pingqing FU | Tianjin University, Institute of Atmospheric Physics, Chinese Academy of Sciences | China |
| Wei HU | Tianjin University | China |
| Chunhui LI | Tongji University | China |
| Bin ZHANG | Xi'an Jiaotong University | China |
| Hongmei XU | Xi'an Jiaotong University, Chinese Academy of Sciences | China |
| Jian SUN | Xi'an Jiaotong University | China |
| Ru-Jin HUANG | Xi'an Jiaotong University, Chinese Academy of Sciences | China |
| Ting WANG | Xi'an Jiaotong University | China |
| Xinyi NIU | Xi'an Jiaotong University, School of Basic Medical Sciences | China |
| Zhenxing SHEN | Xi'an Jiaotong University, School of Basic Medical Sciences | China |
| Dantong LIU | Zhejiang University | China |
| Weijun LI | Zhejiang University of Technology | China |
| Yuanchen CHEN | Zhejiang University of Technology | China |
| František HOPAN | VSB – Technical University of Ostrava | Czech Republic |
| Helena RACLAVSKÁ | VSB – Technical University of Ostrava | Czech Republic |
| Jiří HORÁK | VSB – Technical University of Ostrava | Czech Republic |
| Jiří RYŠAVÝ | VSB – Technical University of Ostrava | Czech Republic |
| Kamil KRPEC | VSB – Technical University of Ostrava | Czech Republic |
| Marek KUCBEL | VSB – Technical University of Ostrava | Czech Republic |
| Jørgen BRANDT | Aarhus University | Denmark |
| Niko KARVOSENOJA | Finnish Environment Institute | Finland |
| Jorma MÄKI-PAAKKANEN | Finnish Institute for Health and Welfare | Finland |
| Ari LESKINEN | Finnish Meteorological Institute | Finland |
| Petri TIITTA | Finnish Meteorological Institute | Finland |
| Risto HILLAMO | Finnish Meteorological Institute | Finland |
| Hanna KOPONEN | University of Eastern Finland | Finland |
| Heikki LAMBERG | University of Eastern Finland | Finland |
| Jarkko TISSARI | University of Eastern Finland | Finland |
| Jorma JOKINIEMI | University of Eastern Finland | Finland |
| Maija-Riitta HIRVONEN | University of Eastern Finland | Finland |
| Miika KORTELAINEN | University of Eastern Finland | Finland |
| Mika IHALAINEN | University of Eastern Finland | Finland |
| Mikko S. HAPPO | University of Eastern Finland | Finland |
| Olli SIPPULA | University of Eastern Finland | Finland |
| Pasi I. JALAVA | University of Eastern Finland | Finland |
| Pasi YLI-PIRILÄ | University of Eastern Finland | Finland |
| Stefanie KASURINEN | University of Eastern Finland | Finland |
| Brice TEMIME-ROUSSEL | Aix-Marseille University, French National Centre for Scientific Research (CNRS) | France |
| Henri WORTHAM | Aix-Marseille University, French National Centre for Scientific Research (CNRS) | France |
| Nicolas MARCHAND | Aix-Marseille University, French National Centre for Scientific Research (CNRS) | France |
| Olivier FAVEZ | French National Institute for Industrial Environment and Risks (Ineris) | France |
| Jean-Luc JAFFREZO | University Grenoble Alpes | France |
| Gontrand LEYSSENS | University of Upper Alsace | France |
| Gwenaëlle TROUVÉ | University of Upper Alsace | France |
| Mejdi JEGUIRIM | University of Upper Alsace | France |
| Gülcin ABBASZADE | Helmholtz Center Munich German Research Center for Environmental Health | Germany |
| Hendryk CZECH | Helmholtz Center Munich German Research Center for Environmental Health | Germany |
| Jürgen ORASCHE | Helmholtz Center Munich German Research Center for Environmental Health | Germany |
| Jürgen SCHNELLE-KREIS | Helmholtz Center Munich German Research Center for Environmental Health | Germany |
| Martin SKLORZ | Helmholtz Center Munich German Research Center for Environmental Health | Germany |
| Ralf ZIMMERMANN | Helmholtz Center Munich German Research Center for Environmental Health, University of Rostock | Germany |
| Thorsten STREIBEL | Helmholtz Center Munich German Research Center for Environmental Health | Germany |
| Claudia SCHÖN | Technology and Support Centre in the Centre of Excellence for Renewable Resources (TFZ) | Germany |
| Hans HARTMANN | Technology and Support Centre in the Centre of Excellence for Renewable Resources (TFZ) | Germany |
| Stefania SQUIZZATO | Institute of Chemical Engineering Sciences | Greece |
| Evangelia DIAPOULI | National Centre for Scientific Research (DEMOKRITOS) | Greece |
| Konstantinos ELEFTHERIADIS | National Centre for Scientific Research (DEMOKRITOS) | Greece |
| Aikaterini BOUGIATIOTI | National Observatory of Athens | Greece |
| Despina PARASKEVOPOULOU | National Observatory of Athens | Greece |
| Dimitrios G. KASKAOUTIS | National Observatory of Athens, University of Western Macedonia | Greece |
| Eleni LIAKAKOU | National Observatory of Athens | Greece |
| Evangelos GERASOPOULOS | National Observatory of Athens | Greece |
| Georgios GRIVAS | National Observatory of Athens | Greece |
| Iasonas STAVROULAS | National Observatory of Athens | Greece |
| Maria TSAGKARAKI | University of Crete | Greece |
| Nikolaos MIHALOPOULOS | University of Crete | Greece |
| Umesh Chandra DUMKA | Aryabhatta Research Institute of Observational Sciences, Graphic Era Deemed to be University | India |
| Atul Kumar SRIVASTAVA | Indian Institute of Tropical Meteorology, Indian Agricultural Research Institute (ICAR) | India |
| Deewan Singh BISHT | Indian Institute of Tropical Meteorology | India |
| Suresh TIWARI | Indian Institute of Tropical Meteorology | India |
| Darius CEBURNIS | University of Galway | Ireland |
| Jurgita OVADNEVAITE | University of Galway | Ireland |
| Cinzia PERRINO | C.N.R. Institute of Atmospheric Pollution Research | Italy |
| Mauro MASIOL | Ca' Foscari University of Venice | Italy |
| Lorenzo MASSIMI | Sapienza University of Rome | Italy |
| Maria Luisa ASTOLFI | Sapienza University of Rome | Italy |
| Silvia CANEPARI | Sapienza University of Rome | Italy |
| Maria Chiara PIETROGRANDE | University of Ferrara | Italy |
| Kimitaka KAWAMURA | Chubu University | Japan |
| Vadimas DUDOITIS | Center for Physical Sciences and Technology (FTMC) | Lithuania |
| Cátia GONÇALVES | University of Aveiro | Portugal |
| Célia ALVES | University of Aveiro | Portugal |
| Estela D. VICENTE | University of Aveiro | Portugal |
| Luís A.C. TARELHO | University of Aveiro | Portugal |
| Margarita EVTYUGINA | University of Aveiro | Portugal |
| Teresa NUNES | University of Aveiro | Portugal |
| Luka DRINOVEC | Institute Jožef Stefan, University of Nova Gorica | Slovenia |
| Asta GREGORIČ | University of Nova Gorica | Slovenia |
| Javier ROYO | University of Zaragoza | Spain |
| Örjan GUSTAFSSON | Stockholm University | Sweden |
| Roger WESTERHOLM | Stockholm University | Sweden |
| Christoffer BOMAN | Umeå University | Sweden |
| Oskari USKI | Umeå University | Sweden |
| Robin NYSTRÖM | Umeå University | Sweden |
| Francesco CANONACO | Datalystica Ltd., Paul Scherrer Institute | Switzerland |
| Amelie BERTRAND | Paul Scherrer Institute | Switzerland |
| André S.H. PRÉVÔT | Paul Scherrer Institute | Switzerland |
| Emily Anne BRUNS | Paul Scherrer Institute | Switzerland |
| Giulia STEFENELLI | Paul Scherrer Institute | Switzerland |
| Imad EL HADDAD | Paul Scherrer Institute | Switzerland |
| Jay Gates SLOWIK | Paul Scherrer Institute | Switzerland |
| Josef DOMMEN | Paul Scherrer Institute | Switzerland |
| Urs BALTENSPERGER | Paul Scherrer Institute | Switzerland |
| Sönke SZIDAT | University of Bern | Switzerland |
| Roy M. HARRISON | University of Birmingham | United Kingdom |
| Zongbo SHI | University of Birmingham | United Kingdom |
| Alan WILLIAMS | University of Leeds | United Kingdom |
| E.J.S. MITCHELL | University of Leeds | United Kingdom |
| Jenny Marie JONES | University of Leeds | United Kingdom |
| Amanda R. LEA-LANGTON | University of Manchester | United Kingdom |
| Philip K. HOPKE | Clarkson University | United States |
| Alexander LASKIN | College of Science, Purdue University | United States |
| Judith C. CHOW | Desert Research Institute | United States |
| John G. WATSON | Desert Research Institute | United States |
| Steven Sai Hang HO | Desert Research Institute | United States |
| George ALLEN | Northeast States for Coordinated Air Use Management | United States |
| Lisa RECTOR | Northeast States for Coordinated Air Use Management | United States |
| Simone M. PIEBER | University of California | United States |
| Curtis W. NOONAN | University of Montana | United States |
| Tony J. WARD | University of Montana | United States |
| Reto GIERÉ | University of Pennsylvania | United States |
